# 1566. Determinants associated with weight gain among persons with HIV who switch to integrase strand transfer inhibitors (INSTI)

**DOI:** 10.1093/ofid/ofad500.1401

**Published:** 2023-11-27

**Authors:** Melissa Klein Cutshaw, Mahmoud Harding, Clemontina A Davenport, Nwora Lance Okeke

**Affiliations:** Duke University, Durham, North Carolina; Duke University, Durham, North Carolina; Duke University, Durham, North Carolina; Duke University, Durham, North Carolina

## Abstract

**Background:**

Weight gain associated with integrase strand transfer inhibitors (INSTI) has been well documented among patients with HIV. However, recent reports suggest that the observed weight gain among patients who switch to INSTIs may be associated in part with their pre-switch regimen.

**Methods:**

We conducted retrospective analyses of a 50% sample of all treatment-experienced patients who switched to second-generation INSTI containing regimen (bictegravir/dolutegravir) at the Duke Adult Infectious Diseases Clinic (Durham, NC, USA) between 1 January 2014 and 31 December 2021. Covariates included sex at birth, age, race, ethnicity, pre-switch regimen, body mass index (BMI), CD4 count and HIV-1 viral load. All weights documented within 24 months from regimen switch were included. Outcomes of interest were percent change in weight and absolute weight change. Ordinary least squares was used to obtain average percent weight change from baseline, adjusted for covariates.

**Results:**

Overall, 339 persons were included in the analyses. Cohort demographics were: median age 51.2 (interquartile range 44.4, 57.6), 73% male sex at birth, 56% Black, 4% Hispanic ethnicity. At regimen switch, median CD4 count was 671 (IQR 460-935), and 78% had a viral load < 200 copies/cc. Mean weight at regimen switch was 87.8 kg (standard deviation [SD] 22.1kg), and mean total weight change over 24 months post-switch was +1.3 kg (SD 7.1 kg). Overall, 28% of cohort members gained >5% of baseline body weight within 24 months of switch. Hispanic ethnicity (5.9% [95% CI 1.0, 10.7]) and BMI < 20 (3.7% [95% CI -0.3, 7.7]) were associated with post-switch weight gain. Only efavirenz-containing pre-switch regimens were significantly associated with weight gain over 24 months post-switch; EFV/FTC/TDF (3.1% [95% CI 0.2, 6.1]), all other EFV-containing regimens (6.3% [95% 0.1-12.4]).

**Conclusion:**

Pre-switch regimens containing efavirenz were significantly associated with weight gain after switch to second-generation INSTI-based regimens, consistent with findings from non-US based cohorts.

**Disclosures:**

**Nwora Lance Okeke, MD MPH**, Gilead Sciences: Advisor/Consultant

